# Supplementary material for: Spatial Distribution and Biochemical Characterization of Serine Peptidase Inhibitors in the Venom of the Brazilian Sea Anemone Anthopleura cascaia Using Mass Spectrometry Imaging
Source: Mar Drugs. 2023 Aug 30;21(9):481. doi: 10.3390/md21090481 (PMC10532579; doi:10.3390/md21090481)

**Figure S1. SDS-PAGE analysis of soluble fraction (SL) of the venom and precipitate fraction of the venom (P).** Samples (20µL) were analysed by SDS-PAGE (12 %) and stained with Comassie brilliant blue. The venom possesses a wide variety of proteins ranging from 14 to 97kDa. **B- Trypsin Inhibition Assay.** The venom (15 µl) was preincubated for 30 min with trypsin (1:1), and afterwards substrate was added to the samples. Substrate consumption in Arbitrary Units of Fluorescence (Y axis) was read every 5 min for 35 min (X axis). In the test, the sea anemone *A. cascaia* venom; Blank (B + S); and Positive Control (E + S), were analysed. B = Buffer, S = Substrate and E = Enzyme. The venom shows the presence of inhibitor components by complete inhibition of Trypsin activity. **C- MALDI-TOF analysis of *A. cascaia's* venom by positive mode**. The mass spectrum shows that *A. cascaia* venom is also composed by low molecular mass components ranging from m/z 3018 to 9995.7.

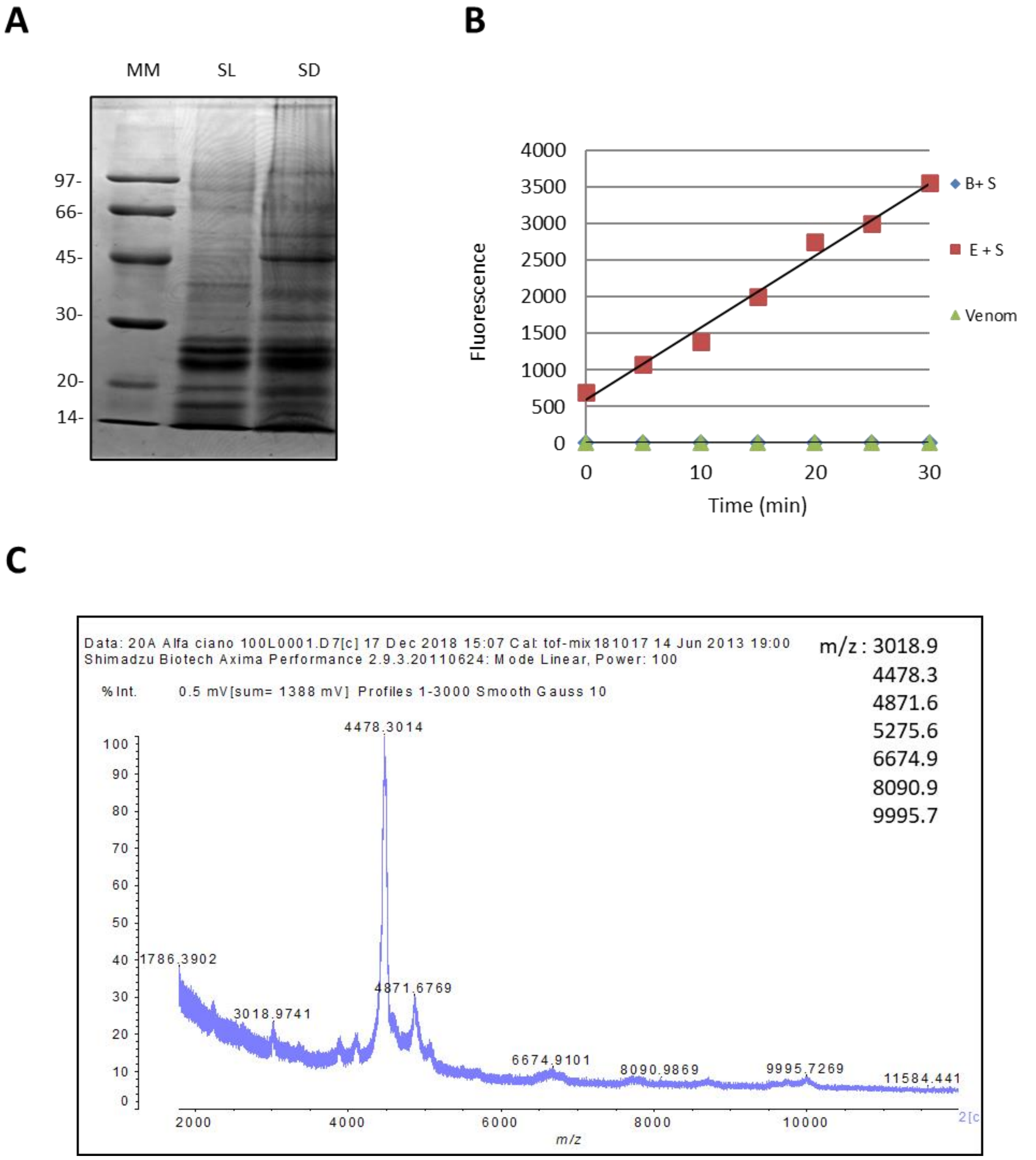

**Figure S2. Trypsin Inhibition Assay.** Fractions (5 to 15 µl) were preincubated for 30 min with trypsin (1:1), and afterwards substrate was added to the samples. Substrate consumption in Arbitrary Units of Fluorescence (Y axis) was read every 5 min for 35 min (X axis). In the test, samples representing venom fractions from *A. cascaia*; Blank (B + S); and Positive Control (E + S), were analysed. B = Buffer, S = Substrate and E = Enzyme. **A- Shows the graph and resulting table of the inhibition assay performed with fractions (F1-F6) from the venom.** F3 and F4 were the only fractions capable of inhibiting trypsin, presenting 100% (F3) and 28% (F4) of inhibition. **B- Shows the graph and resulting table of the inhibition assay performed with fractions from F3.** The subfractions F3.7 and F3.8 presented complete inhibition (100%) of the enzyme activity. **C- Shows the graph of the assay performed with fractions from F3.7.** The subfractions F3.7.5 and 3.7.6 presented 90% of inhibition of the enzymatic activity. **D- Shows the graph of the assay performed with fractions from F3.8.** A wide variety of subfractions presented inhibitory activity, however F3.8.4; F 3.8.6; F3.8.8 were considered the best candidates due to the quality of isolation of peaks, that exhibited 85%; 65% and 59 % of trypsin inhibition, respectively.

**A**

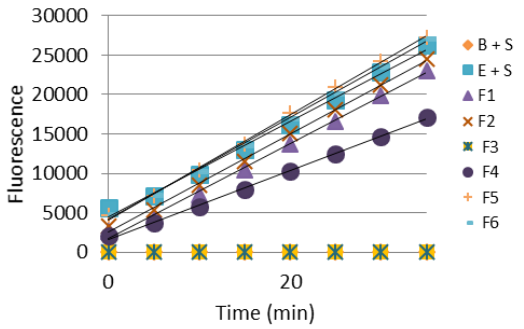


| Sample | Enzyme activity (%) | Inhibition (%) |
|---|---|---|
| Control (E+S) | 100 | 0 |
| F1 | 99 | 1 |
| F2 | 100 | 0 |
| F3 | 0 | 100 |
| F4 | 72 | 28 |
| F5 | 100 | 0 |
| F6 | 100 | 0 |

**B**

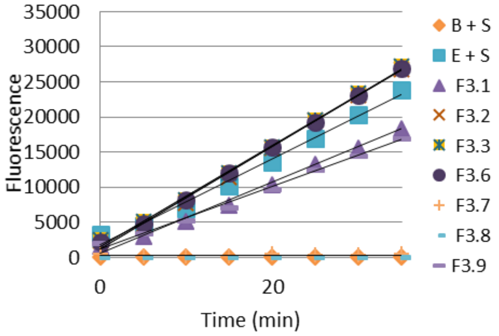


| Sample | Enzyme activity (%) | Inhibition (%) |
|---|---|---|
| Control (E+S) | 100 | 0 |
| F3.1 | 83 | 17 |
| F3.2 | 100 | 0 |
| F3.3 | 100 | 0 |
| F3.4 | 100 | 0 |
| F3.5 | 100 | 0 |
| F3.6 | 100 | 0 |
| F3.7 | 0 | 100 |
| F3.8 | 0 | 100 |
| F3.9 | 72 | 28 |

**C**

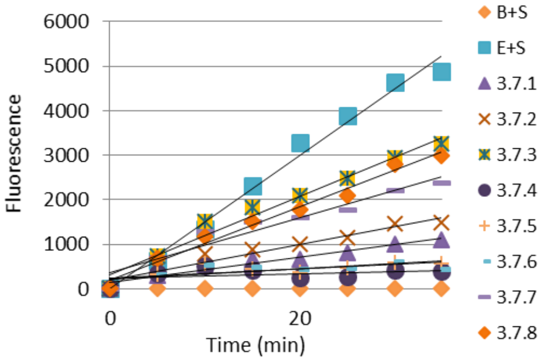


**D**

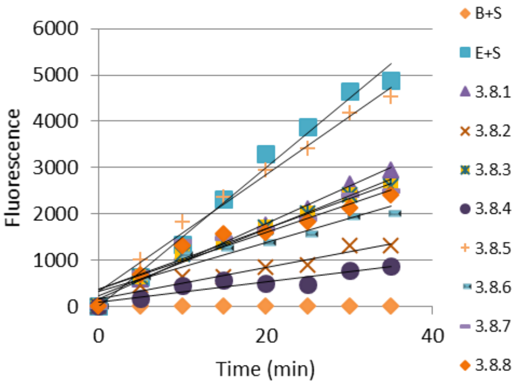

**Figure S3.** Coverage of amino acid sequence from Blastp hit PI-actitoxin-Aeq3a-like based on peptides identified by LC-MS/MS in ACPI-I isolated from *A. cascaia* venom. Supporting peptides R.YYYDESIGTC(+57.02)R.Q and R.QFIFGGC(+57.02)QGNENNFETM(+15.99)K.E are highlighted in grey color and underlined in blue. Cysteine (C) residues present +57.02 mass shift due to alkylation with iodoacetamide and Methionine (M) residues from peptides also present a mass increase of 15.99 due to oxidation. The mass spectra and the error (da) corresponding to the sequence 'YYYDESIGTC' is presented below the sequence.

## ACPI-I analysis by LC-MS/MS

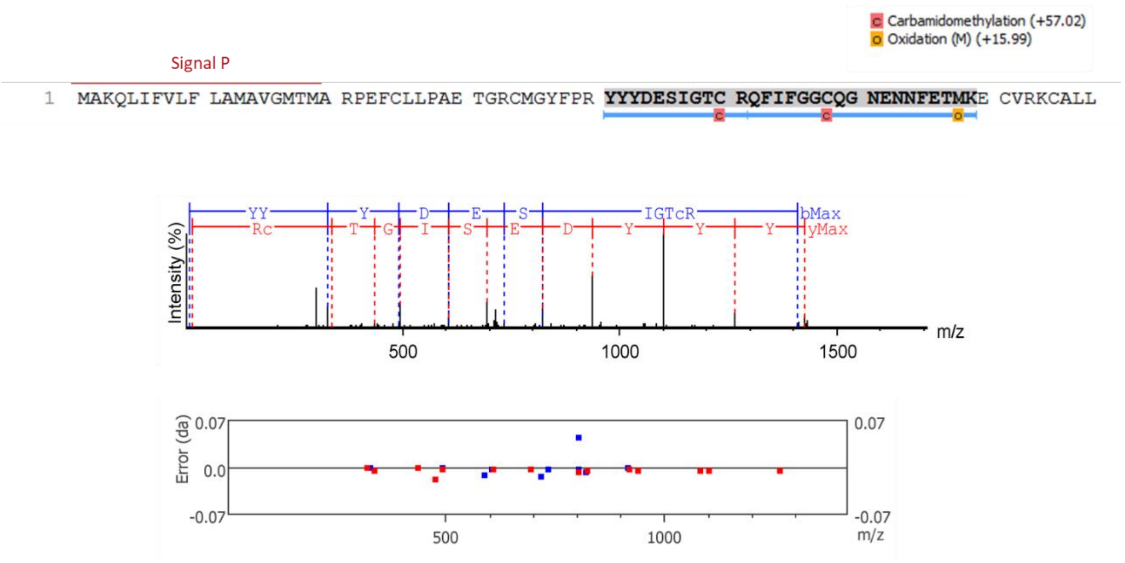

**Figure S4.** Coverage of amino acid sequence from the Blastp hit PI-actitoxin-Aeq3a-like based on peptides identified by LC-MS/MS in ACPI-II isolated from *A. cascaia* venom. Supporting peptides – R.C(+57.02)M(+15.99)GYFPR.Y, R.YYYDESIGTC(+57.02)R.Q and R.QFIFGGC(+57.02)QGNENNFETMK.E – are highlighted in grey color and underlined in blue color. Cysteine (C) residues present +57.02 mass shift due to alkylation with iodoacetamide and Methionine (M) residues also present a mass increase of 15.99 due to oxidation. The mass spectra and the error (da) corresponding to the sequence 'YYYDESIGTC' is presented below the sequence.

## ACPI-II analysis by LC-MS/MS

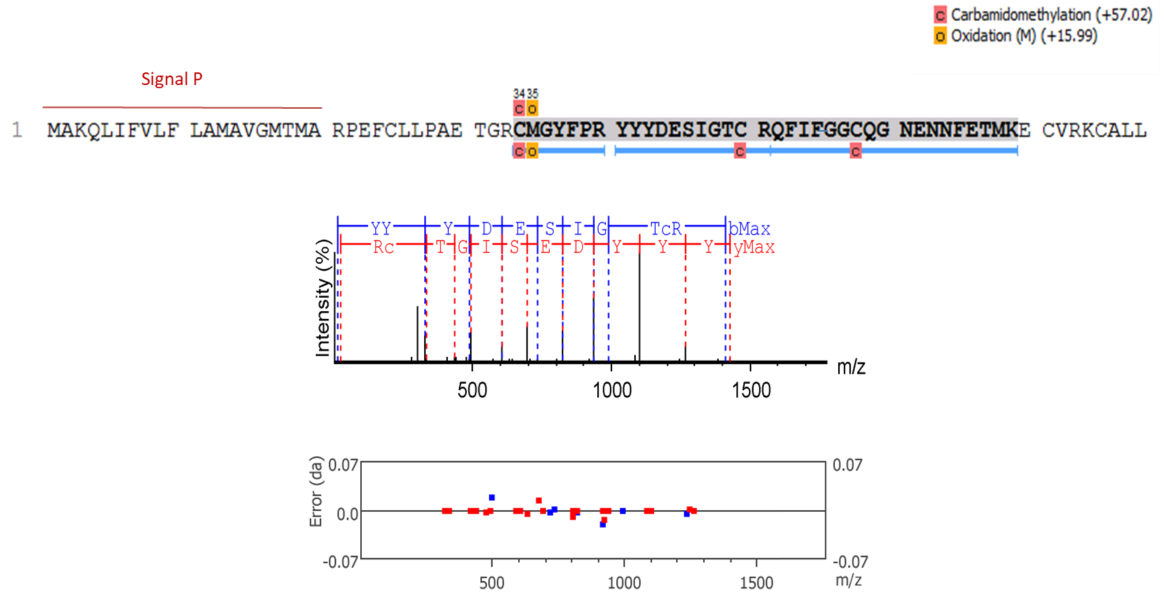

**Figure S5.** Coverage of amino acid sequence from Blastp hit PI-actitoxin-Aeq3a-like based on peptides identified by LC-MS/MS in ACPI-III isolated from *A. cascaia* venom. The supporting peptide –R.YYYDESIGTC(+57.02)R.Q – is highlighted in grey color and underlined in blue color. Cysteine (C) residues present +57.02 mass shift due to alkylation with iodoacetamide. The mass spectra and the error (da) corresponding to the sequence 'YYYDESIGTC' is presented below the sequence.

## ACPI-III analysis by LC-MS/MS

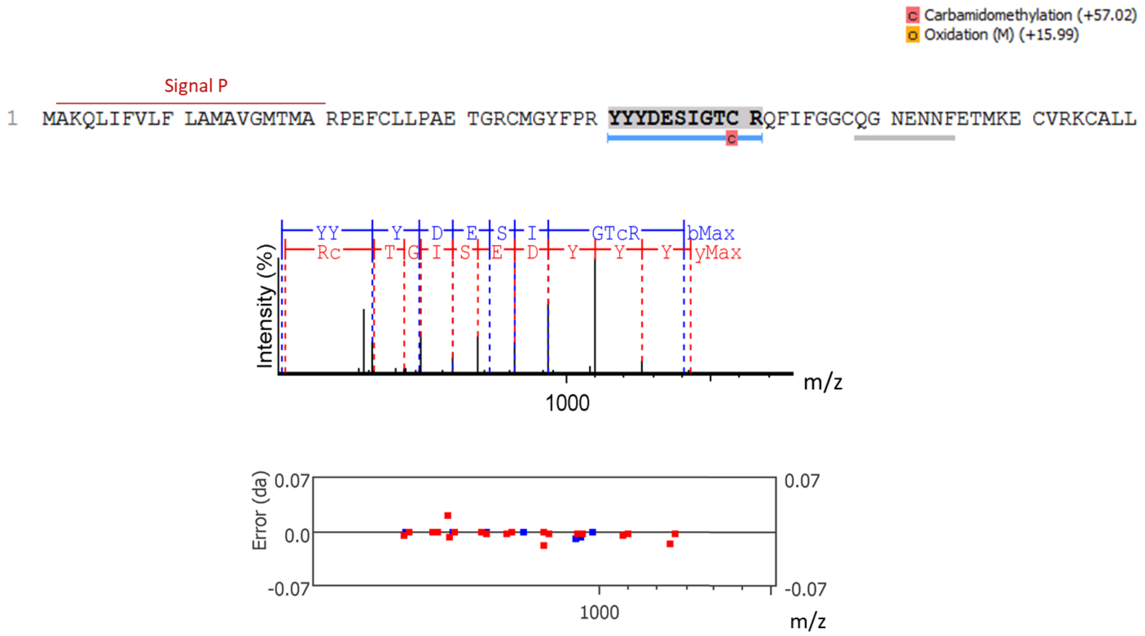

**Table S1.** List of de novo peptides identified by LC-MS/MS in each sample (ACPI-I, ACPI-II and ACPI-III). The table shows the Average Local Confidence percentage (ALC %) for each peptide identified. De novo sequences were submitted to BLASTp and searched using the non-redundant proteins database and Cnidaria taxid at NCBI platform. De novo sequences revealed that peptides from ACPI-I, ACPI-II and ACPI-III present 87%; 78% and 66% of identity to the peptides found in PI-actitoxin-Aeq3a-like from *Actinia tenebrosa,* respectively.

<table>
<tr><th>Isolated peptide</th><th>De novo peptides</th><th>ALC (%)</th><th>E value</th><th>Per ID (%)</th><th rowspan="7">PI-actitoxin-Aeq3a-like from Actinia tenebrosa</th></tr>
<tr><td>ACPI-I</td><td>YYYDESLGHM (+15,99)ER</td><td>73%</td><td>1.2</td><td>87.5</td></tr>
<tr><td rowspan="3">ACPI-II</td><td>DLDM(+15.99)GYFPR</td><td>75%</td><td rowspan="3">2e-13</td><td rowspan="3">77.7</td></tr>
<tr><td>YYYDESLGM (+15.99) M (+15.99) M (+15.99) K</td><td>91%</td></tr>
<tr><td>QDKYGGCQGNENNFETMK</td><td>89%</td></tr>
<tr><td rowspan="2">ACPI-III</td><td>YYYDESLGGSC(+57.02)HK</td><td>71%</td><td rowspan="2">3e-08</td><td rowspan="2">65.62</td></tr>
<tr><td>GYWQNC(+57.02)QGNENNFETMK</td><td>69%</td></tr>
</table>

**Figure S6.** Mass spectra from de novo peptides identified by LC-MS/MS in ACPI-I, ACPI-II and ACPI-III isolated from *A. cascaia* venom.

**ACPI-I** YYYDESLGHMER

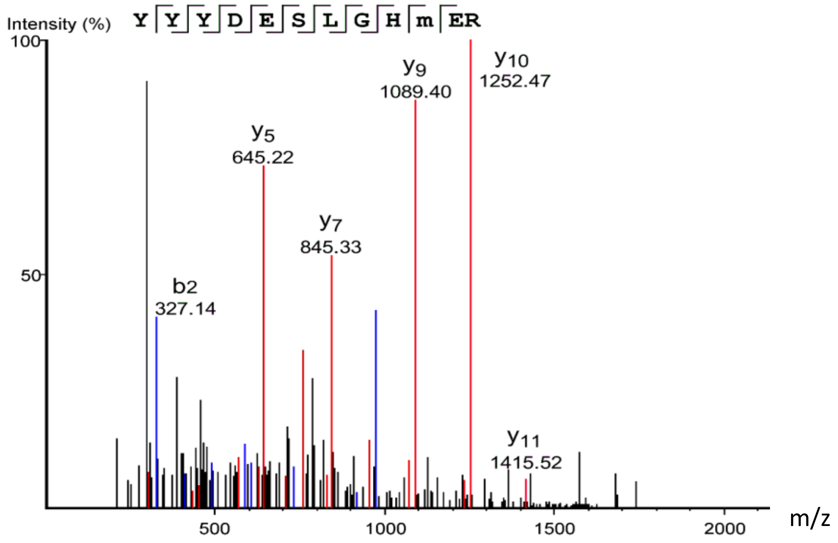


Supplementary material S6 continue in next page.

**ACPI-II** DLDMGYFPR YYYDESLGMMMK QDKYGGCQG NENNFETMK

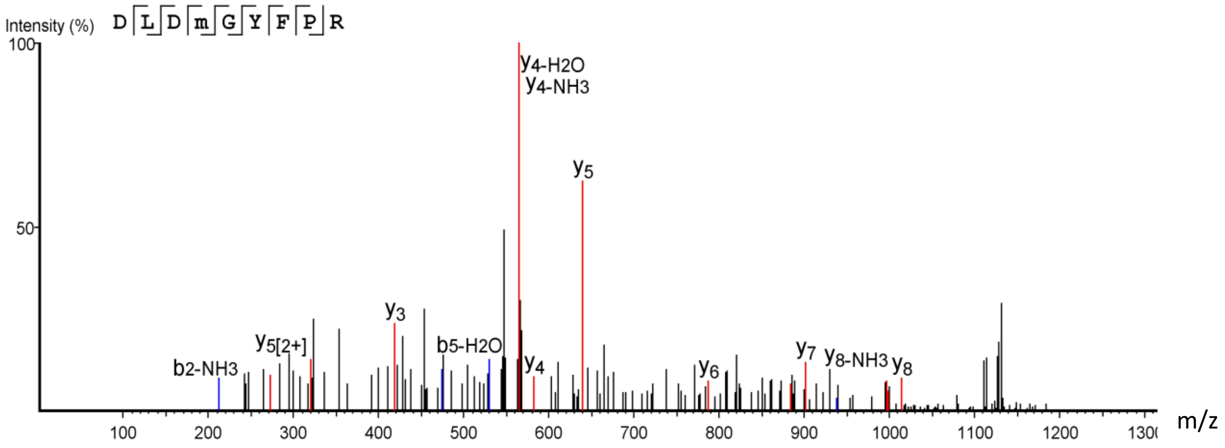


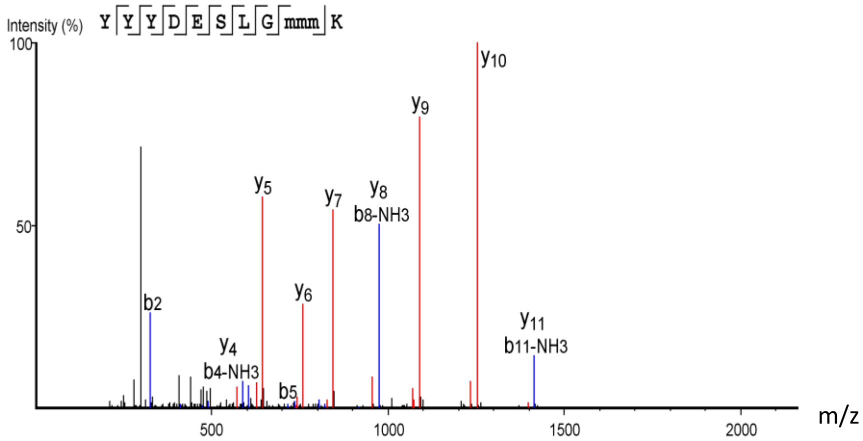


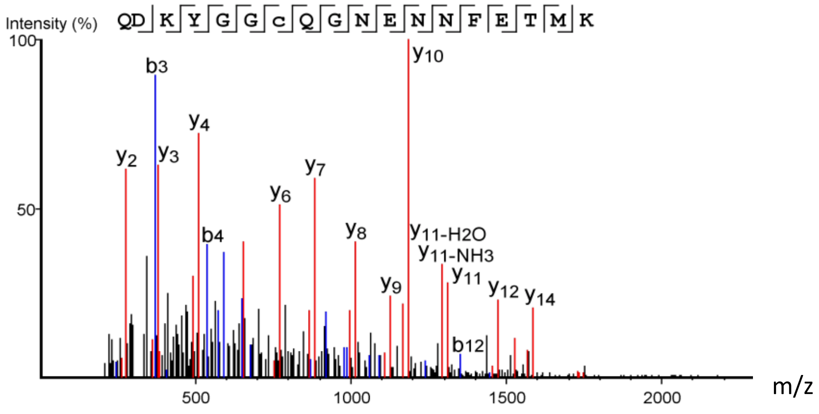


Supplementary material S6 continue in next page.

**ACPI-III**

YYYDESLGGSC GYWQNCQGHK NENNFETMK

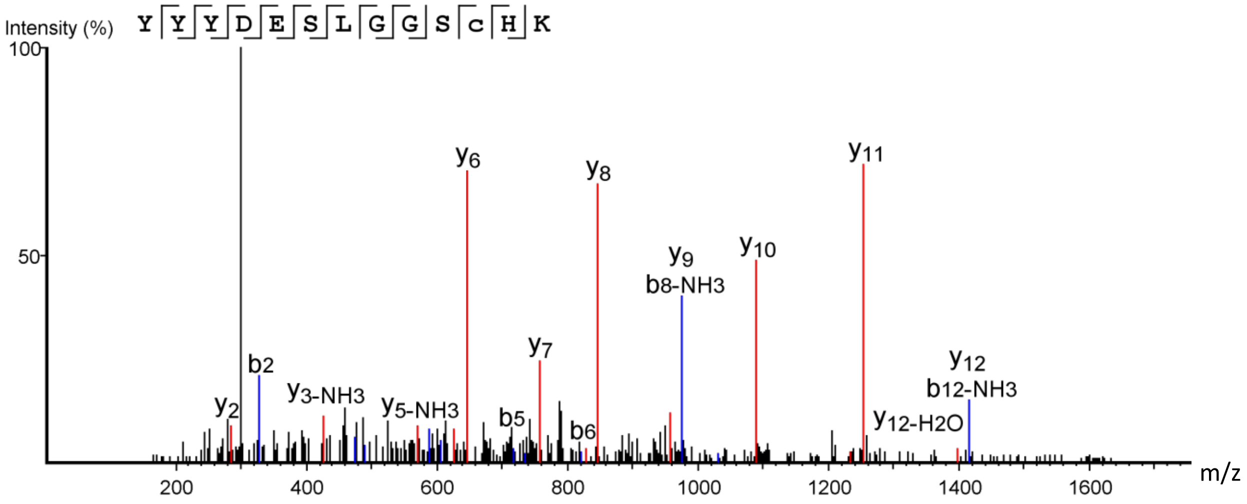


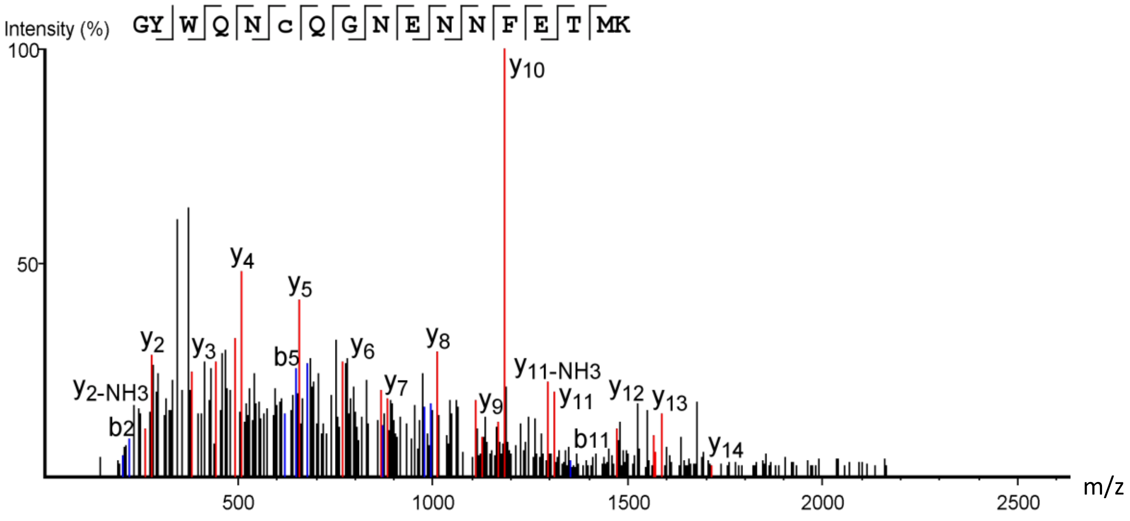

Supplement: Supplementary file 1 [file marinedrugs-21-00481-s001.zip › marinedrugs-2542993-supplementary.pdf]
